# Tetra­kis(μ-2-phen­oxy­propionato)-κ^3^ *O*,*O*′:*O*′;κ^3^ *O*:*O*,*O*′;κ^4^ *O*:*O*′-bis­[(1,10-phenanthroline-κ^2^ *N*,*N*′)(2-phen­oxy­propionato-κ^2^ *O*,*O*′)holmium(III)]

**DOI:** 10.1107/S160053681103738X

**Published:** 2011-09-20

**Authors:** Jin-Bei Shen, Jia-Lu Liu, Guo-Liang Zhao

**Affiliations:** a College of Chemistry and Life Sciences, Zhejiang Normal University, Jinhua 321004, Zhejiang, People’s Republic of China; b Zhejiang Normal University Xingzhi College, Jinhua, Zhejiang 321004, People’s Republic of China

## Abstract

The title compound, [Ho_2_(C_9_H_9_O_3_)_6_(C_12_H_8_N_2_)_2_], lies about a centre of symmetry and is comprised of six 2-phen­oxy­propionate (POPA) anions and two 1,10-phenanthroline (phen) ligands. The two Ho^III^ ions are linked by four POPA groups utilizing both bi- and tridentate bridging modes to form an inversion-symmetric dimer. Each Ho^III^ ion is nine-coordinate, with a chelating 1,10-phenanthroline mol­ecule, one bidentate chelating carboxyl­ate group, two bidentate bridging carboxyl­ate groups and two tridentate bridging carboxyl­ate groups in a distorted mono-capped square anti­prism geometry. There are weak π–π aromatic inter­actions between the phen groups and aromatic rings of the POPA ligands [centroid–centroid distance = 3.829 (1) Å].

## Related literature

For phen­oxy­alkanoic acids, see: Markus & Buser (1997 ▶). For holmium complexes, see: Hu *et al.* (2006 ▶); Zhao *et al.* (2010 ▶). For isotypic complexes, see: Shen *et al.* (2011*a* ▶,*b* ▶,*c* ▶,*d* ▶).
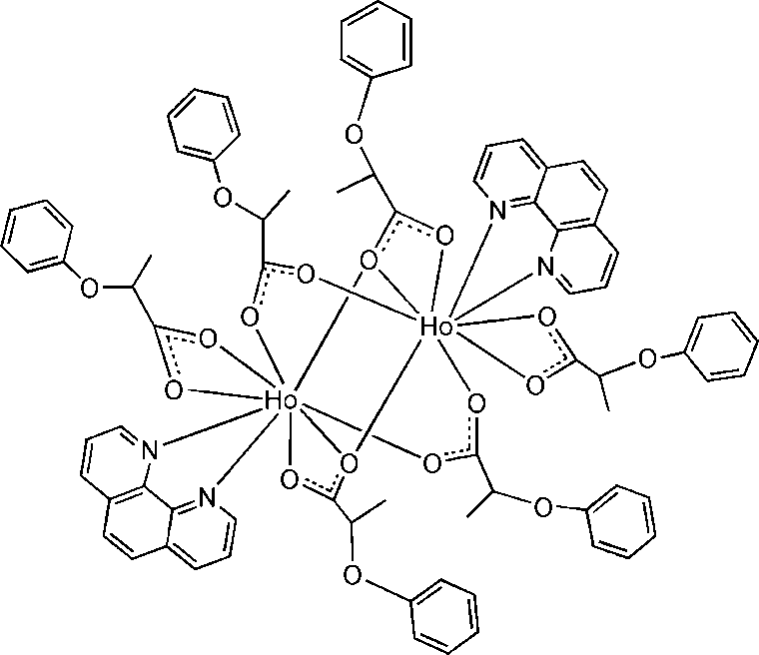


## Experimental

### 

#### Crystal data

[Ho_2_(C_9_H_9_O_3_)_6_(C_12_H_8_N_2_)_2_]
*M* *_r_* = 1681.24
Monoclinic, 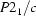

*a* = 11.4657 (2) Å
*b* = 25.7960 (3) Å
*c* = 13.8366 (2) Å
β = 120.687 (1)°
*V* = 3519.37 (9) Å^3^
*Z* = 2
Mo *K*α radiation
μ = 2.31 mm^−1^
*T* = 296 K
0.25 × 0.16 × 0.06 mm

#### Data collection

Bruker APEXII area-detector diffractometer
Absorption correction: multi-scan (*SADABS*; Sheldrick, 1996 ▶) *T*_min_ = 0.656, *T*_max_ = 0.877
24604 measured reflections
6189 independent reflections
4266 reflections with *I* > 2σ(*I*)
*R*_int_ = 0.053

#### Refinement

*R*[*F* ^2^ > 2σ(*F* ^2^)] = 0.033
*wR*(*F* ^2^) = 0.062
*S* = 1.00
6189 reflections
464 parameters
H-atom parameters constrained
Δρ_max_ = 0.67 e Å^−3^
Δρ_min_ = −0.55 e Å^−3^

### 

Data collection: *APEX2* (Bruker, 2006 ▶); cell refinement: *SAINT* (Bruker, 2006 ▶); data reduction: *SAINT*; program(s) used to solve structure: *SHELXS97* (Sheldrick, 2008 ▶); program(s) used to refine structure: *SHELXL97* (Sheldrick, 2008 ▶); molecular graphics: *SHELXTL* (Sheldrick, 2008 ▶); software used to prepare material for publication: *SHELXL97*.

## Supplementary Material

Crystal structure: contains datablock(s) global. DOI: 10.1107/S160053681103738X/pk2341sup1.cif

Additional supplementary materials:  crystallographic information; 3D view; checkCIF report

## Figures and Tables

**Fig. 1.**
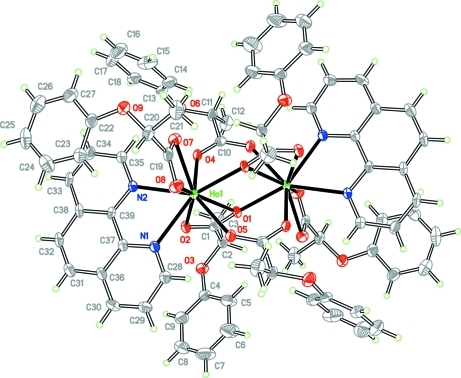
The molecular structure of the title complex. Displacement ellipsoids are drawn at the 30% probability level.

**Fig. 2.**
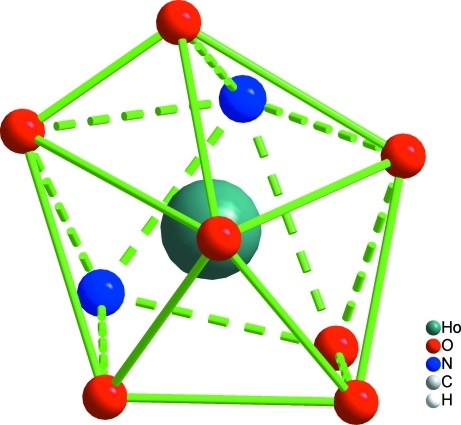
The coordination environment of the HoIII.

## supplementary crystallographic information

### Comment

The group of phenoxyalkanoic acids includes a considerable number of important herbicides.  The desired biological activity is largely dependent on the length of the carbon chain of the alkanoic acid, the nature of the phenoxy group, and the position of its attachment to the carbon chain (Markus & Buser, 1997).  The fascinating structures of 2-phenoxypropionic acid complexes coupled with their special functionality catch our interests.  Recently, we have reported our partial research results (Shen *et al.*, 2011*a*,*b*,*c*,*d*). Here we describe a new Ho^III^ complex.

The structure of the complex is shown in Fig. 1 and the coordination environment of Ho^III^ is shown in Fig. 2.  The dimeric title compound is centrosymmetric and is comprised of six *L* and two phen ligands. The two Ho^III^ ions are linked together by four *L* groups by bi- and tridentate bridging modes to form a dimeric unit with crystallographic inversion symmetry. The distance between two Ho^III^ ions is 3.9769 (4) Å, which is similar to analogous complexes (Hu *et al.*, 2006; Zhao *et al.*, 2010).  Each Ho^III^ ion is coordinated to nine atoms, of which five are oxygen atoms from bridging carboxylates, two are oxygen atoms from the bidentate chelating carboxylate groups, and two are nitrogen atoms from a 1,10-phenanthroline molecule. The analysis of structural features indicates that the central Ho^III^ ion adopts a distorted mono-capped square antiprism geometry (Fig. 2). The Ho—O distances are all within the range 2.312 (3)–2.618 (3) Å, and the Ho—N distances are 2.500 (3) and 2.594 (3) Å.  There are weak π–π aromatic interactions from phen molecules and aromatic rings of the *L* ligands.

### Experimental

Reagents and solvents were of commercially available quality and were used without further purification.  2-phenoxypropionic acid (1.5 mmol), Ho(NO_3_)_3_.6H_2_O (0.5 mmol) and 1,10-phenanthroline (0.5 mmol) were dissolved in 20 ml ethanol, then 10 ml water was added.  The mixed solution was stirred for 12 h at room temperature.  Solid deposits were removed by filtration, and the colourless solution allowed to slowly evaporate in air. Colourless crystals were obtained after several days.

### Refinement

The structure was solved by direct methods and successive Fourier difference maps.  The H atoms bonded to C and N atoms were positioned geometrically and refined using a riding model [aliphatic C—H =0.96 Å (*U*_iso_(H) = 1.5*U*_eq_(C)), aromatic C—H = 0.93 Å (*U*_iso_(H) = 1.2*U*_eq_(C))].

### Crystal data

**Table:**

[Ho_2_(C_9_H_9_O_3_)_6_(C_12_H_8_N_2_)_2_]	*F*(000) = 1688
*M**_r_* = 1681.24	*D*_x_ = 1.587 Mg m^−^^3^
Monoclinic, *P*2_1_/*c*	Mo *K*α radiation, λ = 0.71073 Å
Hall symbol: -P 2ybc	Cell parameters from 4156 reflections
*a* = 11.4657 (2) Å	θ = 1.6–25.0°
*b* = 25.7960 (3) Å	µ = 2.31 mm^−^^1^
*c* = 13.8366 (2) Å	*T* = 296 K
β = 120.687 (1)°	Block, colourless
*V* = 3519.37 (9) Å^3^	0.25 × 0.16 × 0.06 mm
*Z* = 2	

### Data collection

**Table:**

Bruker APEXII area-detector diffractometer	6189 independent reflections
Radiation source: fine-focus sealed tube	4266 reflections with *I* > 2σ(*I*)
graphite	*R*_int_ = 0.053
φ and ω scans	θ_max_ = 25.0°, θ_min_ = 1.6°
Absorption correction: multi-scan (*SADABS*; Sheldrick, 1996)	*h* = −13→10
*T*_min_ = 0.656, *T*_max_ = 0.877	*k* = −30→30
24604 measured reflections	*l* = −16→16

### Refinement

**Table:**

Refinement on *F*^2^	Primary atom site location: structure-invariant direct methods
Least-squares matrix: full	Secondary atom site location: difference Fourier map
*R*[*F*^2^ > 2σ(*F*^2^)] = 0.033	Hydrogen site location: inferred from neighbouring sites
*wR*(*F*^2^) = 0.062	H-atom parameters constrained
*S* = 1.00	*w* = 1/[σ^2^(*F*_o_^2^) + (0.0194*P*)^2^] where *P* = (*F*_o_^2^ + 2*F*_c_^2^)/3
6189 reflections	(Δ/σ)_max_ = 0.001
464 parameters	Δρ_max_ = 0.67 e Å^−^^3^
0 restraints	Δρ_min_ = −0.55 e Å^−^^3^

### Special details

**Table:**

Geometry. All e.s.d.'s (except the e.s.d. in the dihedral angle between two l.s. planes) are estimated using the full covariance matrix. The cell e.s.d.'s are taken into account individually in the estimation of e.s.d.'s in distances, angles and torsion angles; correlations between e.s.d.'s in cell parameters are only used when they are defined by crystal symmetry. An approximate (isotropic) treatment of cell e.s.d.'s is used for estimating e.s.d.'s involving l.s. planes.
Refinement. Refinement of *F*^2^ against ALL reflections. The weighted *R*-factor *wR* and goodness of fit *S* are based on *F*^2^, conventional *R*-factors *R* are based on *F*, with *F* set to zero for negative *F*^2^. The threshold expression of *F*^2^ > σ(*F*^2^) is used only for calculating *R*-factors(gt) *etc*. and is not relevant to the choice of reflections for refinement. *R*-factors based on *F*^2^ are statistically about twice as large as those based on *F*, and *R*- factors based on ALL data will be even larger.

### Fractional atomic coordinates and isotropic or equivalent isotropic displacement parameters (Å^2^)

**Table:**

	*x*	*y*	*z*	*U*_iso_*/*U*_eq_	
Ho1	0.453322 (18)	−0.002635 (6)	0.840539 (15)	0.02616 (7)	
O1	0.3839 (3)	0.03107 (9)	0.9827 (2)	0.0317 (7)	
O2	0.2468 (3)	0.04173 (9)	0.8017 (2)	0.0334 (7)	
O3	0.0920 (3)	0.11048 (9)	0.8413 (2)	0.0406 (8)	
O4	0.3347 (3)	−0.06690 (9)	0.8774 (2)	0.0315 (7)	
O5	0.5809 (3)	0.06950 (9)	0.9358 (2)	0.0385 (8)	
O6	0.1723 (3)	−0.15132 (11)	0.8456 (3)	0.0537 (9)	
O7	0.5312 (3)	−0.08474 (9)	0.8029 (2)	0.0393 (8)	
O8	0.6179 (3)	−0.01225 (9)	0.7835 (3)	0.0427 (8)	
O9	0.5943 (3)	−0.13350 (10)	0.6562 (3)	0.0516 (9)	
N1	0.3815 (3)	0.06779 (11)	0.6866 (3)	0.0317 (8)	
N2	0.2831 (3)	−0.03033 (11)	0.6449 (3)	0.0303 (8)	
C1	0.2748 (4)	0.04761 (13)	0.9009 (4)	0.0293 (10)	
C2	0.1741 (4)	0.07333 (14)	0.9257 (4)	0.0348 (11)	
H2	0.2229	0.0904	0.9991	0.052*	
C3	0.0773 (5)	0.03325 (16)	0.9260 (4)	0.0511 (13)	
H3A	0.0131	0.0500	0.9406	0.077*	
H3B	0.1277	0.0080	0.9835	0.077*	
H3C	0.0300	0.0164	0.8542	0.077*	
C4	0.1569 (5)	0.15377 (15)	0.8342 (4)	0.0401 (12)	
C5	0.2863 (5)	0.16791 (17)	0.9097 (5)	0.076 (2)	
H5	0.3398	0.1469	0.9715	0.091*	
C6	0.3385 (7)	0.2135 (2)	0.8946 (6)	0.102 (2)	
H6	0.4262	0.2234	0.9481	0.123*	
C7	0.2635 (7)	0.24415 (19)	0.8027 (6)	0.088 (2)	
H7	0.3004	0.2742	0.7919	0.105*	
C8	0.1341 (7)	0.22997 (19)	0.7274 (5)	0.0779 (19)	
H8	0.0814	0.2510	0.6654	0.094*	
C9	0.0793 (5)	0.18452 (17)	0.7415 (5)	0.0618 (15)	
H9	−0.0090	0.1749	0.6888	0.074*	
C10	0.3458 (4)	−0.08535 (14)	0.9658 (4)	0.0313 (11)	
C11	0.2612 (5)	−0.13343 (15)	0.9561 (4)	0.0384 (12)	
H11	0.3240	−0.1616	0.9984	0.058*	
C12	0.1759 (5)	−0.12281 (17)	1.0082 (4)	0.0574 (14)	
H12A	0.1252	−0.1533	1.0032	0.086*	
H12B	0.1145	−0.0948	0.9689	0.086*	
H12C	0.2337	−0.1135	1.0857	0.086*	
C13	0.2213 (6)	−0.18197 (14)	0.7927 (4)	0.0466 (13)	
C14	0.3536 (6)	−0.18429 (15)	0.8191 (4)	0.0537 (14)	
H14	0.4204	−0.1658	0.8799	0.064*	
C15	0.3859 (7)	−0.21419 (19)	0.7548 (6)	0.082 (2)	
H15	0.4755	−0.2156	0.7718	0.098*	
C16	0.2897 (10)	−0.2419 (2)	0.6662 (6)	0.100 (3)	
H16	0.3138	−0.2624	0.6238	0.121*	
C17	0.1569 (9)	−0.2397 (2)	0.6396 (5)	0.094 (2)	
H17	0.0907	−0.2583	0.5786	0.113*	
C18	0.1218 (6)	−0.20985 (16)	0.7032 (5)	0.0650 (16)	
H18	0.0323	−0.2084	0.6862	0.078*	
C19	0.6011 (5)	−0.06062 (16)	0.7727 (4)	0.0369 (11)	
C20	0.6725 (5)	−0.09027 (16)	0.7207 (4)	0.0440 (12)	
H20	0.6894	−0.0669	0.6733	0.066*	
C21	0.8065 (5)	−0.11263 (18)	0.8140 (4)	0.0668 (16)	
H21A	0.8502	−0.1313	0.7812	0.100*	
H21B	0.7891	−0.1357	0.8597	0.100*	
H21C	0.8642	−0.0849	0.8596	0.100*	
C22	0.4850 (5)	−0.12432 (17)	0.5496 (4)	0.0447 (12)	
C23	0.4492 (5)	−0.07704 (18)	0.4982 (4)	0.0508 (13)	
H23	0.4958	−0.0473	0.5362	0.061*	
C24	0.3431 (6)	−0.0744 (2)	0.3893 (5)	0.0618 (15)	
H24	0.3197	−0.0424	0.3532	0.074*	
C25	0.2704 (5)	−0.1175 (2)	0.3321 (5)	0.0665 (16)	
H25	0.1985	−0.1151	0.2585	0.080*	
C26	0.3070 (6)	−0.1646 (2)	0.3868 (5)	0.0679 (17)	
H26	0.2585	−0.1942	0.3498	0.081*	
C27	0.4133 (5)	−0.16837 (17)	0.4946 (5)	0.0557 (14)	
H27	0.4372	−0.2003	0.5306	0.067*	
C28	0.4299 (5)	0.11540 (14)	0.7059 (4)	0.0415 (12)	
H28	0.5047	0.1229	0.7760	0.050*	
C29	0.3747 (5)	0.15526 (15)	0.6267 (4)	0.0509 (14)	
H29	0.4124	0.1883	0.6440	0.061*	
C30	0.2652 (5)	0.14510 (15)	0.5242 (4)	0.0486 (13)	
H30	0.2272	0.1712	0.4705	0.058*	
C31	0.0953 (5)	0.08180 (17)	0.3942 (4)	0.0459 (13)	
H31	0.0526	0.1072	0.3394	0.055*	
C32	0.0474 (5)	0.03298 (17)	0.3718 (4)	0.0419 (12)	
H32	−0.0276	0.0253	0.3019	0.050*	
C33	0.0655 (4)	−0.05879 (16)	0.4337 (4)	0.0401 (12)	
H33	−0.0076	−0.0686	0.3644	0.048*	
C34	0.1309 (5)	−0.09425 (16)	0.5172 (4)	0.0425 (12)	
H34	0.1041	−0.1288	0.5047	0.051*	
C35	0.2385 (4)	−0.07875 (14)	0.6218 (4)	0.0372 (11)	
H35	0.2808	−0.1036	0.6779	0.045*	
C36	0.2098 (5)	0.09545 (15)	0.4997 (4)	0.0365 (11)	
C37	0.2728 (4)	0.05725 (14)	0.5842 (4)	0.0295 (10)	
C38	0.1101 (4)	−0.00725 (15)	0.4539 (3)	0.0359 (10)	
C39	0.2193 (4)	0.00509 (14)	0.5603 (3)	0.0292 (9)	

### Atomic displacement parameters (Å^2^)

**Table:**

	*U*^11^	*U*^22^	*U*^33^	*U*^12^	*U*^13^	*U*^23^
Ho1	0.02474 (12)	0.02897 (10)	0.02041 (12)	−0.00023 (10)	0.00836 (9)	−0.00045 (9)
O1	0.0256 (19)	0.0364 (15)	0.0211 (18)	0.0048 (13)	0.0032 (16)	0.0027 (13)
O2	0.0289 (19)	0.0441 (16)	0.0230 (19)	0.0058 (13)	0.0103 (17)	0.0018 (13)
O3	0.0237 (18)	0.0361 (15)	0.049 (2)	0.0042 (13)	0.0094 (17)	0.0010 (14)
O4	0.0348 (19)	0.0339 (14)	0.0211 (18)	−0.0086 (13)	0.0108 (16)	−0.0037 (13)
O5	0.045 (2)	0.0368 (15)	0.027 (2)	−0.0107 (14)	0.0129 (18)	−0.0025 (14)
O6	0.045 (2)	0.0491 (18)	0.052 (3)	−0.0090 (16)	0.014 (2)	−0.0099 (16)
O7	0.033 (2)	0.0384 (15)	0.044 (2)	0.0008 (14)	0.0175 (18)	−0.0031 (14)
O8	0.048 (2)	0.0388 (17)	0.050 (2)	−0.0029 (14)	0.0315 (19)	−0.0047 (14)
O9	0.061 (2)	0.0438 (17)	0.040 (2)	0.0085 (16)	0.018 (2)	−0.0067 (16)
N1	0.032 (2)	0.0333 (18)	0.026 (2)	−0.0017 (16)	0.012 (2)	0.0012 (16)
N2	0.028 (2)	0.0331 (17)	0.021 (2)	−0.0006 (16)	0.0064 (19)	−0.0003 (16)
C1	0.027 (3)	0.026 (2)	0.032 (3)	−0.0072 (19)	0.013 (3)	−0.002 (2)
C2	0.031 (3)	0.039 (2)	0.029 (3)	0.004 (2)	0.012 (2)	0.000 (2)
C3	0.048 (3)	0.062 (3)	0.059 (4)	−0.003 (2)	0.039 (3)	0.000 (3)
C4	0.030 (3)	0.035 (2)	0.045 (4)	0.006 (2)	0.012 (3)	−0.002 (2)
C5	0.043 (4)	0.044 (3)	0.084 (5)	−0.010 (3)	−0.010 (4)	0.016 (3)
C6	0.071 (5)	0.065 (4)	0.111 (6)	−0.023 (3)	0.002 (5)	0.017 (4)
C7	0.077 (5)	0.047 (3)	0.115 (6)	−0.010 (3)	0.032 (5)	0.016 (4)
C8	0.087 (5)	0.055 (3)	0.067 (5)	0.004 (3)	0.021 (4)	0.017 (3)
C9	0.052 (4)	0.048 (3)	0.060 (4)	0.004 (3)	0.011 (3)	0.002 (3)
C10	0.031 (3)	0.029 (2)	0.037 (3)	0.0013 (19)	0.020 (3)	−0.001 (2)
C11	0.040 (3)	0.040 (2)	0.030 (3)	−0.015 (2)	0.013 (3)	−0.007 (2)
C12	0.052 (4)	0.072 (3)	0.056 (4)	−0.019 (3)	0.033 (3)	−0.010 (3)
C13	0.070 (4)	0.023 (2)	0.047 (4)	−0.002 (2)	0.030 (3)	−0.005 (2)
C14	0.060 (4)	0.037 (3)	0.062 (4)	−0.002 (3)	0.029 (4)	−0.005 (2)
C15	0.110 (6)	0.048 (3)	0.116 (6)	0.007 (3)	0.079 (5)	0.004 (3)
C16	0.177 (9)	0.065 (4)	0.086 (6)	0.007 (5)	0.086 (7)	−0.012 (4)
C17	0.135 (7)	0.058 (4)	0.057 (5)	−0.005 (5)	0.026 (5)	−0.022 (3)
C18	0.073 (4)	0.036 (3)	0.059 (4)	−0.002 (3)	0.014 (4)	−0.001 (3)
C19	0.030 (3)	0.048 (3)	0.024 (3)	0.002 (2)	0.007 (2)	−0.005 (2)
C20	0.039 (3)	0.055 (3)	0.036 (3)	0.000 (2)	0.018 (3)	−0.010 (2)
C21	0.045 (4)	0.096 (4)	0.051 (4)	0.027 (3)	0.018 (3)	−0.012 (3)
C22	0.048 (4)	0.054 (3)	0.037 (3)	0.008 (3)	0.025 (3)	−0.005 (3)
C23	0.050 (4)	0.056 (3)	0.042 (4)	−0.001 (3)	0.021 (3)	−0.004 (3)
C24	0.054 (4)	0.082 (4)	0.053 (4)	0.010 (3)	0.030 (4)	0.019 (3)
C25	0.041 (4)	0.118 (5)	0.033 (4)	−0.003 (4)	0.014 (3)	−0.010 (4)
C26	0.059 (4)	0.075 (4)	0.068 (5)	−0.014 (3)	0.031 (4)	−0.026 (3)
C27	0.053 (4)	0.049 (3)	0.059 (4)	0.001 (3)	0.024 (3)	−0.013 (3)
C28	0.042 (3)	0.040 (2)	0.035 (3)	−0.002 (2)	0.014 (3)	0.001 (2)
C29	0.059 (4)	0.035 (2)	0.049 (4)	−0.004 (2)	0.020 (3)	0.007 (2)
C30	0.052 (4)	0.040 (3)	0.050 (4)	0.009 (2)	0.023 (3)	0.018 (2)
C31	0.047 (3)	0.058 (3)	0.032 (3)	0.015 (3)	0.020 (3)	0.012 (2)
C32	0.034 (3)	0.063 (3)	0.020 (3)	0.008 (2)	0.008 (3)	−0.001 (2)
C33	0.033 (3)	0.058 (3)	0.021 (3)	−0.004 (2)	0.007 (3)	−0.012 (2)
C34	0.045 (3)	0.045 (2)	0.032 (3)	−0.013 (2)	0.016 (3)	−0.010 (2)
C35	0.038 (3)	0.039 (2)	0.030 (3)	−0.001 (2)	0.014 (3)	−0.001 (2)
C36	0.037 (3)	0.045 (2)	0.027 (3)	0.010 (2)	0.016 (3)	0.007 (2)
C37	0.024 (3)	0.040 (2)	0.026 (3)	0.008 (2)	0.014 (2)	0.001 (2)
C38	0.028 (3)	0.056 (3)	0.018 (3)	−0.001 (2)	0.008 (2)	−0.002 (2)
C39	0.028 (2)	0.039 (2)	0.021 (2)	−0.001 (2)	0.013 (2)	−0.006 (2)

### Geometric parameters (Å, °)

**Table:**

Ho1—O1^i^	2.312 (3)	C12—H12B	0.9600
Ho1—O5	2.318 (3)	C12—H12C	0.9600
Ho1—O4	2.359 (3)	C13—C14	1.368 (6)
Ho1—O8	2.401 (3)	C13—C18	1.382 (6)
Ho1—O2	2.431 (3)	C14—C15	1.364 (7)
Ho1—O7	2.455 (2)	C14—H14	0.9300
Ho1—N2	2.500 (3)	C15—C16	1.361 (8)
Ho1—N1	2.594 (3)	C15—H15	0.9300
Ho1—O1	2.618 (3)	C16—C17	1.373 (8)
Ho1—C19	2.759 (4)	C16—H16	0.9300
Ho1—C1	2.883 (4)	C17—C18	1.374 (8)
Ho1—Ho1^i^	3.9769 (4)	C17—H17	0.9300
O1—C1	1.258 (5)	C18—H18	0.9300
O1—Ho1^i^	2.312 (3)	C19—C20	1.541 (5)
O2—C1	1.249 (5)	C20—C21	1.528 (6)
O3—C4	1.373 (5)	C20—H20	0.9800
O3—C2	1.430 (4)	C21—H21A	0.9600
O4—C10	1.257 (5)	C21—H21B	0.9600
O5—C10^i^	1.249 (5)	C21—H21C	0.9600
O6—C13	1.377 (5)	C22—C23	1.365 (6)
O6—C11	1.414 (5)	C22—C27	1.381 (6)
O7—C19	1.243 (5)	C23—C24	1.372 (6)
O8—C19	1.260 (4)	C23—H23	0.9300
O9—C22	1.384 (5)	C24—C25	1.374 (6)
O9—C20	1.424 (5)	C24—H24	0.9300
N1—C28	1.318 (4)	C25—C26	1.377 (7)
N1—C37	1.354 (5)	C25—H25	0.9300
N2—C35	1.325 (4)	C26—C27	1.365 (6)
N2—C39	1.366 (5)	C26—H26	0.9300
C1—C2	1.515 (5)	C27—H27	0.9300
C2—C3	1.518 (5)	C28—C29	1.397 (5)
C2—H2	0.9800	C28—H28	0.9300
C3—H3A	0.9600	C29—C30	1.356 (6)
C3—H3B	0.9600	C29—H29	0.9300
C3—H3C	0.9600	C30—C36	1.392 (5)
C4—C5	1.358 (6)	C30—H30	0.9300
C4—C9	1.379 (6)	C31—C32	1.346 (5)
C5—C6	1.382 (6)	C31—C36	1.422 (6)
C5—H5	0.9300	C31—H31	0.9300
C6—C7	1.367 (7)	C32—C38	1.432 (5)
C6—H6	0.9300	C32—H32	0.9300
C7—C8	1.357 (7)	C33—C34	1.360 (5)
C7—H7	0.9300	C33—C38	1.401 (5)
C8—C9	1.391 (6)	C33—H33	0.9300
C8—H8	0.9300	C34—C35	1.398 (5)
C9—H9	0.9300	C34—H34	0.9300
C10—O5^i^	1.249 (5)	C35—H35	0.9300
C10—C11	1.538 (5)	C36—C37	1.413 (5)
C11—C12	1.507 (6)	C37—C39	1.445 (5)
C11—H11	0.9800	C38—C39	1.398 (5)
C12—H12A	0.9600		
			
O1^i^—Ho1—O5	73.65 (9)	C6—C7—H7	120.6
O1^i^—Ho1—O4	77.96 (9)	C7—C8—C9	121.0 (5)
O5—Ho1—O4	135.01 (9)	C7—C8—H8	119.5
O1^i^—Ho1—O8	88.25 (10)	C9—C8—H8	119.5
O5—Ho1—O8	84.04 (9)	C4—C9—C8	119.5 (5)
O4—Ho1—O8	129.41 (8)	C4—C9—H9	120.2
O1^i^—Ho1—O2	123.55 (9)	C8—C9—H9	120.2
O5—Ho1—O2	90.48 (9)	O5^i^—C10—O4	127.9 (4)
O4—Ho1—O2	76.97 (9)	O5^i^—C10—C11	113.7 (4)
O8—Ho1—O2	144.74 (9)	O4—C10—C11	118.3 (4)
O1^i^—Ho1—O7	76.49 (9)	O6—C11—C12	106.9 (4)
O5—Ho1—O7	128.40 (10)	O6—C11—C10	115.6 (4)
O4—Ho1—O7	75.63 (9)	C12—C11—C10	110.3 (3)
O8—Ho1—O7	53.80 (9)	O6—C11—H11	107.9
O2—Ho1—O7	141.07 (9)	C12—C11—H11	107.9
O1^i^—Ho1—N2	144.81 (9)	C10—C11—H11	107.9
O5—Ho1—N2	139.68 (10)	C11—C12—H12A	109.5
O4—Ho1—N2	79.38 (10)	C11—C12—H12B	109.5
O8—Ho1—N2	85.69 (10)	H12A—C12—H12B	109.5
O2—Ho1—N2	76.18 (10)	C11—C12—H12C	109.5
O7—Ho1—N2	72.01 (10)	H12A—C12—H12C	109.5
O1^i^—Ho1—N1	146.90 (10)	H12B—C12—H12C	109.5
O5—Ho1—N1	75.43 (10)	C14—C13—O6	125.5 (4)
O4—Ho1—N1	133.91 (10)	C14—C13—C18	120.8 (5)
O8—Ho1—N1	77.03 (10)	O6—C13—C18	113.7 (5)
O2—Ho1—N1	67.89 (10)	C15—C14—C13	119.0 (5)
O7—Ho1—N1	115.29 (10)	C15—C14—H14	120.5
N2—Ho1—N1	64.27 (10)	C13—C14—H14	120.5
O1^i^—Ho1—O1	72.63 (10)	C16—C15—C14	121.3 (6)
O5—Ho1—O1	69.61 (9)	C16—C15—H15	119.3
O4—Ho1—O1	68.97 (8)	C14—C15—H15	119.3
O8—Ho1—O1	150.74 (9)	C15—C16—C17	119.8 (7)
O2—Ho1—O1	51.26 (9)	C15—C16—H16	120.1
O7—Ho1—O1	136.78 (9)	C17—C16—H16	120.1
N2—Ho1—O1	122.64 (10)	C16—C17—C18	120.0 (6)
N1—Ho1—O1	107.07 (9)	C16—C17—H17	120.0
O1^i^—Ho1—C19	83.31 (11)	C18—C17—H17	120.0
O5—Ho1—C19	108.06 (12)	C17—C18—C13	119.2 (6)
O4—Ho1—C19	102.38 (12)	C17—C18—H18	120.4
O8—Ho1—C19	27.15 (10)	C13—C18—H18	120.4
O2—Ho1—C19	151.44 (11)	O7—C19—O8	122.8 (4)
O7—Ho1—C19	26.78 (10)	O7—C19—C20	119.7 (4)
N2—Ho1—C19	75.67 (11)	O8—C19—C20	117.5 (4)
N1—Ho1—C19	95.34 (12)	O7—C19—Ho1	62.9 (2)
O1—Ho1—C19	155.57 (11)	O8—C19—Ho1	60.4 (2)
O1^i^—Ho1—C1	98.36 (12)	C20—C19—Ho1	173.1 (3)
O5—Ho1—C1	79.01 (10)	O9—C20—C21	105.9 (4)
O4—Ho1—C1	71.37 (9)	O9—C20—C19	111.5 (4)
O8—Ho1—C1	159.21 (9)	C21—C20—C19	109.7 (4)
O2—Ho1—C1	25.40 (10)	O9—C20—H20	109.9
O7—Ho1—C1	146.93 (10)	C21—C20—H20	109.9
N2—Ho1—C1	99.48 (12)	C19—C20—H20	109.9
N1—Ho1—C1	87.04 (11)	C20—C21—H21A	109.5
O1—Ho1—C1	25.86 (10)	C20—C21—H21B	109.5
C19—Ho1—C1	172.89 (12)	H21A—C21—H21B	109.5
O1^i^—Ho1—Ho1^i^	38.93 (6)	C20—C21—H21C	109.5
O5—Ho1—Ho1^i^	66.86 (7)	H21A—C21—H21C	109.5
O4—Ho1—Ho1^i^	69.05 (6)	H21B—C21—H21C	109.5
O8—Ho1—Ho1^i^	123.82 (7)	C23—C22—C27	120.8 (5)
O2—Ho1—Ho1^i^	84.79 (7)	C23—C22—O9	125.2 (4)
O7—Ho1—Ho1^i^	110.11 (7)	C27—C22—O9	114.0 (4)
N2—Ho1—Ho1^i^	146.12 (7)	C22—C23—C24	118.6 (5)
N1—Ho1—Ho1^i^	132.87 (7)	C22—C23—H23	120.7
O1—Ho1—Ho1^i^	33.70 (6)	C24—C23—H23	120.7
C19—Ho1—Ho1^i^	122.12 (9)	C23—C24—C25	121.9 (5)
C1—Ho1—Ho1^i^	59.47 (9)	C23—C24—H24	119.1
C1—O1—Ho1^i^	162.7 (3)	C25—C24—H24	119.1
C1—O1—Ho1	88.9 (2)	C24—C25—C26	118.2 (5)
Ho1^i^—O1—Ho1	107.37 (10)	C24—C25—H25	120.9
C1—O2—Ho1	98.0 (3)	C26—C25—H25	120.9
C4—O3—C2	116.6 (3)	C27—C26—C25	121.0 (5)
C10—O4—Ho1	133.9 (3)	C27—C26—H26	119.5
C10^i^—O5—Ho1	139.6 (3)	C25—C26—H26	119.5
C13—O6—C11	119.9 (4)	C26—C27—C22	119.4 (5)
C19—O7—Ho1	90.4 (2)	C26—C27—H27	120.3
C19—O8—Ho1	92.4 (3)	C22—C27—H27	120.3
C22—O9—C20	118.1 (3)	N1—C28—C29	123.5 (4)
C28—N1—C37	117.8 (3)	N1—C28—H28	118.3
C28—N1—Ho1	124.2 (3)	C29—C28—H28	118.3
C37—N1—Ho1	117.1 (2)	C30—C29—C28	119.0 (4)
C35—N2—C39	116.8 (4)	C30—C29—H29	120.5
C35—N2—Ho1	121.5 (3)	C28—C29—H29	120.5
C39—N2—Ho1	121.1 (2)	C29—C30—C36	119.7 (4)
O2—C1—O1	121.8 (4)	C29—C30—H30	120.1
O2—C1—C2	120.2 (4)	C36—C30—H30	120.1
O1—C1—C2	117.9 (4)	C32—C31—C36	121.5 (4)
O2—C1—Ho1	56.6 (2)	C32—C31—H31	119.2
O1—C1—Ho1	65.2 (2)	C36—C31—H31	119.2
C2—C1—Ho1	176.6 (3)	C31—C32—C38	121.0 (4)
O3—C2—C1	111.6 (3)	C31—C32—H32	119.5
O3—C2—C3	106.3 (3)	C38—C32—H32	119.5
C1—C2—C3	110.2 (3)	C34—C33—C38	118.8 (4)
O3—C2—H2	109.5	C34—C33—H33	120.6
C1—C2—H2	109.5	C38—C33—H33	120.6
C3—C2—H2	109.5	C33—C34—C35	120.0 (4)
C2—C3—H3A	109.5	C33—C34—H34	120.0
C2—C3—H3B	109.5	C35—C34—H34	120.0
H3A—C3—H3B	109.5	N2—C35—C34	123.2 (4)
C2—C3—H3C	109.5	N2—C35—H35	118.4
H3A—C3—H3C	109.5	C34—C35—H35	118.4
H3B—C3—H3C	109.5	C30—C36—C37	117.6 (4)
C5—C4—O3	125.7 (4)	C30—C36—C31	123.0 (4)
C5—C4—C9	119.6 (5)	C37—C36—C31	119.4 (4)
O3—C4—C9	114.7 (4)	N1—C37—C36	122.3 (4)
C4—C5—C6	120.0 (5)	N1—C37—C39	118.9 (3)
C4—C5—H5	120.0	C36—C37—C39	118.8 (4)
C6—C5—H5	120.0	C39—C38—C33	117.8 (4)
C7—C6—C5	121.2 (6)	C39—C38—C32	119.2 (4)
C7—C6—H6	119.4	C33—C38—C32	123.0 (4)
C5—C6—H6	119.4	N2—C39—C38	123.4 (4)
C8—C7—C6	118.7 (5)	N2—C39—C37	116.6 (4)
C8—C7—H7	120.6	C38—C39—C37	120.0 (4)
			
O1^i^—Ho1—O1—C1	−174.0 (3)	O4—Ho1—C1—O2	−98.9 (2)
O5—Ho1—O1—C1	107.5 (2)	O8—Ho1—C1—O2	79.4 (4)
O4—Ho1—O1—C1	−90.5 (2)	O7—Ho1—C1—O2	−95.1 (3)
O8—Ho1—O1—C1	134.6 (2)	N2—Ho1—C1—O2	−23.6 (2)
O2—Ho1—O1—C1	−0.6 (2)	N1—Ho1—C1—O2	39.7 (2)
O7—Ho1—O1—C1	−127.6 (2)	O1—Ho1—C1—O2	−179.0 (4)
N2—Ho1—O1—C1	−29.2 (2)	Ho1^i^—Ho1—C1—O2	−175.1 (2)
N1—Ho1—O1—C1	40.7 (2)	O1^i^—Ho1—C1—O1	5.8 (2)
C19—Ho1—O1—C1	−163.6 (3)	O5—Ho1—C1—O1	−65.6 (2)
Ho1^i^—Ho1—O1—C1	−174.0 (3)	O4—Ho1—C1—O1	80.1 (2)
O1^i^—Ho1—O1—Ho1^i^	0.0	O8—Ho1—C1—O1	−101.6 (4)
O5—Ho1—O1—Ho1^i^	−78.56 (11)	O2—Ho1—C1—O1	179.0 (4)
O4—Ho1—O1—Ho1^i^	83.47 (11)	O7—Ho1—C1—O1	83.9 (3)
O8—Ho1—O1—Ho1^i^	−51.4 (2)	N2—Ho1—C1—O1	155.4 (2)
O2—Ho1—O1—Ho1^i^	173.40 (14)	N1—Ho1—C1—O1	−141.3 (2)
O7—Ho1—O1—Ho1^i^	46.34 (16)	Ho1^i^—Ho1—C1—O1	3.89 (16)
N2—Ho1—O1—Ho1^i^	144.74 (10)	C4—O3—C2—C1	−66.7 (4)
N1—Ho1—O1—Ho1^i^	−145.30 (10)	C4—O3—C2—C3	173.1 (3)
C19—Ho1—O1—Ho1^i^	10.4 (3)	O2—C1—C2—O3	−30.2 (5)
C1—Ho1—O1—Ho1^i^	174.0 (3)	O1—C1—C2—O3	152.3 (3)
O1^i^—Ho1—O2—C1	8.1 (2)	O2—C1—C2—C3	87.8 (4)
O5—Ho1—O2—C1	−62.5 (2)	O1—C1—C2—C3	−89.8 (4)
O4—Ho1—O2—C1	73.9 (2)	C2—O3—C4—C5	−10.2 (6)
O8—Ho1—O2—C1	−142.8 (2)	C2—O3—C4—C9	170.4 (4)
O7—Ho1—O2—C1	120.1 (2)	O3—C4—C5—C6	−177.9 (5)
N2—Ho1—O2—C1	156.0 (2)	C9—C4—C5—C6	1.5 (9)
N1—Ho1—O2—C1	−136.5 (2)	C4—C5—C6—C7	−2.1 (10)
O1—Ho1—O2—C1	0.6 (2)	C5—C6—C7—C8	2.1 (11)
C19—Ho1—O2—C1	165.9 (3)	C6—C7—C8—C9	−1.5 (10)
Ho1^i^—Ho1—O2—C1	4.2 (2)	C5—C4—C9—C8	−0.9 (8)
O1^i^—Ho1—O4—C10	26.4 (3)	O3—C4—C9—C8	178.6 (4)
O5—Ho1—O4—C10	−25.2 (4)	C7—C8—C9—C4	0.9 (9)
O8—Ho1—O4—C10	104.0 (3)	Ho1—O4—C10—O5^i^	8.8 (7)
O2—Ho1—O4—C10	−102.6 (3)	Ho1—O4—C10—C11	−171.0 (2)
O7—Ho1—O4—C10	105.4 (3)	C13—O6—C11—C12	−155.4 (4)
N2—Ho1—O4—C10	179.3 (4)	C13—O6—C11—C10	81.5 (5)
N1—Ho1—O4—C10	−143.2 (3)	O5^i^—C10—C11—O6	177.4 (4)
O1—Ho1—O4—C10	−49.4 (3)	O4—C10—C11—O6	−2.8 (6)
C19—Ho1—O4—C10	106.7 (3)	O5^i^—C10—C11—C12	56.0 (5)
C1—Ho1—O4—C10	−76.8 (4)	O4—C10—C11—C12	−124.2 (4)
Ho1^i^—Ho1—O4—C10	−13.2 (3)	C11—O6—C13—C14	−22.5 (6)
O1^i^—Ho1—O5—C10^i^	−24.5 (4)	C11—O6—C13—C18	160.5 (4)
O4—Ho1—O5—C10^i^	28.6 (5)	O6—C13—C14—C15	−176.1 (4)
O8—Ho1—O5—C10^i^	−114.4 (4)	C18—C13—C14—C15	0.7 (7)
O2—Ho1—O5—C10^i^	100.5 (4)	C13—C14—C15—C16	−0.6 (8)
O7—Ho1—O5—C10^i^	−81.6 (4)	C14—C15—C16—C17	0.6 (10)
N2—Ho1—O5—C10^i^	169.5 (4)	C15—C16—C17—C18	−0.7 (11)
N1—Ho1—O5—C10^i^	167.5 (4)	C16—C17—C18—C13	0.7 (9)
O1—Ho1—O5—C10^i^	52.7 (4)	C14—C13—C18—C17	−0.8 (8)
C19—Ho1—O5—C10^i^	−101.6 (4)	O6—C13—C18—C17	176.4 (4)
C1—Ho1—O5—C10^i^	77.7 (4)	Ho1—O7—C19—O8	7.8 (4)
Ho1^i^—Ho1—O5—C10^i^	16.4 (4)	Ho1—O7—C19—C20	−172.7 (4)
O1^i^—Ho1—O7—C19	−102.1 (3)	Ho1—O8—C19—O7	−8.0 (4)
O5—Ho1—O7—C19	−46.2 (3)	Ho1—O8—C19—C20	172.5 (3)
O4—Ho1—O7—C19	177.1 (3)	O1^i^—Ho1—C19—O7	73.2 (2)
O8—Ho1—O7—C19	−4.3 (2)	O5—Ho1—C19—O7	143.5 (2)
O2—Ho1—O7—C19	130.5 (2)	O4—Ho1—C19—O7	−2.9 (3)
N2—Ho1—O7—C19	93.8 (3)	O8—Ho1—C19—O7	172.4 (4)
N1—Ho1—O7—C19	44.9 (3)	O2—Ho1—C19—O7	−88.4 (3)
O1—Ho1—O7—C19	−147.4 (2)	N2—Ho1—C19—O7	−78.4 (2)
C1—Ho1—O7—C19	173.3 (3)	N1—Ho1—C19—O7	−140.1 (2)
Ho1^i^—Ho1—O7—C19	−122.1 (2)	O1—Ho1—C19—O7	63.2 (4)
O1^i^—Ho1—O8—C19	78.7 (2)	Ho1^i^—Ho1—C19—O7	70.0 (3)
O5—Ho1—O8—C19	152.5 (3)	O1^i^—Ho1—C19—O8	−99.3 (2)
O4—Ho1—O8—C19	5.9 (3)	O5—Ho1—C19—O8	−28.9 (3)
O2—Ho1—O8—C19	−125.2 (2)	O4—Ho1—C19—O8	−175.4 (2)
O7—Ho1—O8—C19	4.2 (2)	O2—Ho1—C19—O8	99.2 (3)
N2—Ho1—O8—C19	−66.6 (2)	O7—Ho1—C19—O8	−172.4 (4)
N1—Ho1—O8—C19	−131.2 (3)	N2—Ho1—C19—O8	109.2 (3)
O1—Ho1—O8—C19	127.0 (3)	N1—Ho1—C19—O8	47.5 (2)
C1—Ho1—O8—C19	−172.1 (3)	O1—Ho1—C19—O8	−109.3 (3)
Ho1^i^—Ho1—O8—C19	95.5 (2)	Ho1^i^—Ho1—C19—O8	−102.5 (2)
O1^i^—Ho1—N1—C28	−22.1 (4)	C22—O9—C20—C21	−162.5 (4)
O5—Ho1—N1—C28	−0.7 (3)	C22—O9—C20—C19	78.2 (5)
O4—Ho1—N1—C28	139.1 (3)	O7—C19—C20—O9	33.8 (6)
O8—Ho1—N1—C28	−87.8 (3)	O8—C19—C20—O9	−146.8 (4)
O2—Ho1—N1—C28	95.9 (3)	O7—C19—C20—C21	−83.2 (5)
O7—Ho1—N1—C28	−126.6 (3)	O8—C19—C20—C21	96.3 (5)
N2—Ho1—N1—C28	−179.3 (4)	C20—O9—C22—C23	3.4 (6)
O1—Ho1—N1—C28	62.2 (3)	C20—O9—C22—C27	−178.5 (4)
C19—Ho1—N1—C28	−108.0 (3)	C27—C22—C23—C24	−2.0 (7)
C1—Ho1—N1—C28	78.7 (3)	O9—C22—C23—C24	176.0 (4)
Ho1^i^—Ho1—N1—C28	36.6 (4)	C22—C23—C24—C25	1.6 (8)
O1^i^—Ho1—N1—C37	168.8 (2)	C23—C24—C25—C26	−0.3 (8)
O5—Ho1—N1—C37	−169.8 (3)	C24—C25—C26—C27	−0.6 (8)
O4—Ho1—N1—C37	−30.0 (3)	C25—C26—C27—C22	0.2 (8)
O8—Ho1—N1—C37	103.0 (3)	C23—C22—C27—C26	1.2 (7)
O2—Ho1—N1—C37	−73.2 (3)	O9—C22—C27—C26	−177.0 (4)
O7—Ho1—N1—C37	64.2 (3)	C37—N1—C28—C29	0.2 (6)
N2—Ho1—N1—C37	11.6 (2)	Ho1—N1—C28—C29	−168.9 (3)
O1—Ho1—N1—C37	−107.0 (3)	N1—C28—C29—C30	0.2 (7)
C19—Ho1—N1—C37	82.9 (3)	C28—C29—C30—C36	0.1 (7)
C1—Ho1—N1—C37	−90.4 (3)	C36—C31—C32—C38	0.1 (7)
Ho1^i^—Ho1—N1—C37	−132.5 (2)	C38—C33—C34—C35	1.5 (7)
O1^i^—Ho1—N2—C35	18.3 (4)	C39—N2—C35—C34	−0.5 (6)
O5—Ho1—N2—C35	174.6 (3)	Ho1—N2—C35—C34	171.3 (3)
O4—Ho1—N2—C35	−32.4 (3)	C33—C34—C35—N2	−0.9 (7)
O8—Ho1—N2—C35	99.1 (3)	C29—C30—C36—C37	−0.8 (6)
O2—Ho1—N2—C35	−111.4 (3)	C29—C30—C36—C31	−179.9 (4)
O7—Ho1—N2—C35	45.8 (3)	C32—C31—C36—C30	177.5 (4)
N1—Ho1—N2—C35	176.7 (3)	C32—C31—C36—C37	−1.6 (6)
O1—Ho1—N2—C35	−88.8 (3)	C28—N1—C37—C36	−1.0 (6)
C19—Ho1—N2—C35	73.5 (3)	Ho1—N1—C37—C36	168.9 (3)
C1—Ho1—N2—C35	−101.2 (3)	C28—N1—C37—C39	179.0 (4)
Ho1^i^—Ho1—N2—C35	−53.7 (4)	Ho1—N1—C37—C39	−11.2 (4)
O1^i^—Ho1—N2—C39	−170.3 (2)	C30—C36—C37—N1	1.3 (6)
O5—Ho1—N2—C39	−14.0 (4)	C31—C36—C37—N1	−179.6 (4)
O4—Ho1—N2—C39	139.0 (3)	C30—C36—C37—C39	−178.7 (4)
O8—Ho1—N2—C39	−89.5 (3)	C31—C36—C37—C39	0.5 (6)
O2—Ho1—N2—C39	60.0 (3)	C34—C33—C38—C39	−0.8 (6)
O7—Ho1—N2—C39	−142.8 (3)	C34—C33—C38—C32	−179.5 (4)
N1—Ho1—N2—C39	−11.9 (3)	C31—C32—C38—C39	2.6 (6)
O1—Ho1—N2—C39	82.6 (3)	C31—C32—C38—C33	−178.7 (4)
C19—Ho1—N2—C39	−115.1 (3)	C35—N2—C39—C38	1.2 (6)
C1—Ho1—N2—C39	70.2 (3)	Ho1—N2—C39—C38	−170.5 (3)
Ho1^i^—Ho1—N2—C39	117.7 (3)	C35—N2—C39—C37	−177.0 (3)
Ho1—O2—C1—O1	−1.1 (4)	Ho1—N2—C39—C37	11.3 (5)
Ho1—O2—C1—C2	−178.6 (3)	C33—C38—C39—N2	−0.6 (6)
Ho1^i^—O1—C1—O2	−159.3 (6)	C32—C38—C39—N2	178.1 (4)
Ho1—O1—C1—O2	1.0 (4)	C33—C38—C39—C37	177.5 (4)
Ho1^i^—O1—C1—C2	18.2 (10)	C32—C38—C39—C37	−3.7 (6)
Ho1—O1—C1—C2	178.5 (3)	N1—C37—C39—N2	0.5 (5)
Ho1^i^—O1—C1—Ho1	−160.3 (8)	C36—C37—C39—N2	−179.5 (3)
O1^i^—Ho1—C1—O2	−173.2 (2)	N1—C37—C39—C38	−177.8 (3)
O5—Ho1—C1—O2	115.4 (2)	C36—C37—C39—C38	2.2 (6)

Symmetry codes: (i) −*x*+1, −*y*, −*z*+2.

## References

[bb1] Bruker (2006). *APEX2* and *SAINT* Bruker AXS Inc., Madison, Wisconsin, USA.

[bb2] Hu, X.-L., Qiu, L., Sun, W.-B. & Chen, Z. (2006). *Acta Cryst.* E**62**, m3213–m3214.

[bb3] Markus, D. M. & Buser, H. R. (1997). *Environ. Sci. Technol.* **31**, 1953–1959.

[bb4] Sheldrick, G. M. (1996). *SADABS* University of Göttingen, Germany.

[bb5] Sheldrick, G. M. (2008). *Acta Cryst.* A**64**, 112–122. 10.1107/S0108767307043930 18156677

[bb6] Shen, J.-B., Liu, J.-L. & Zhao, G.-L. (2011*a*). *Acta Cryst.* E**67**, m1234. 10.1107/S1600536811032041 PMC3200853 22058858

[bb7] Shen, J.-B., Liu, J.-L. & Zhao, G.-L. (2011*b*). *Acta Cryst.* E**67**, m1319. 10.1107/S1600536811034829 PMC3200638 22064817

[bb8] Shen, J.-B., Liu, J.-L. & Zhao, G.-L. (2011*c*). *Acta Cryst.* E**67**, m1320. 10.1107/S1600536811034696 PMC3200901 22058895

[bb9] Shen, J.-B., Liu, J.-L. & Zhao, G.-L. (2011*d*). *Acta Cryst.* E**67**, m1321. 10.1107/S1600536811034702 PMC3200949 22058896

[bb10] Zhao, G.-L., Liu, J.-L. & Liu, J.-F. (2010). *Acta Cryst.* E**66**, m1272–m1273. 10.1107/S1600536810036408 PMC2983278 21587417

